# Targeting Myeloid Checkpoint Molecules in Combination With Antibody Therapy: A Novel Anti-Cancer Strategy With IgA Antibodies?

**DOI:** 10.3389/fimmu.2022.932155

**Published:** 2022-07-05

**Authors:** Chilam Chan, Marta Lustig, Niklas Baumann, Thomas Valerius, Geert van Tetering, Jeanette H. W. Leusen

**Affiliations:** ^1^ Center for Translational Immunology, University Medical Center Utrecht, Utrecht, Netherlands; ^2^ Division of Stem Cell Transplantation and Immunotherapy, Department of Medicine II, Christian Albrechts University Kiel and University Medical Center Schleswig-Holstein, Kiel, Germany

**Keywords:** IgA, myeloid checkpoints, neutrophils (PMNs), cancer immonotherapy, immune checkpoint, antibodies, CD47-SIRPalpha axis, macrophages

## Abstract

Immunotherapy with therapeutic antibodies has shown a lack of durable responses in some patients due to resistance mechanisms. Checkpoint molecules expressed by tumor cells have a deleterious impact on clinical responses to therapeutic antibodies. Myeloid checkpoints, which negatively regulate macrophage and neutrophil anti-tumor responses, are a novel type of checkpoint molecule. Myeloid checkpoint inhibition is currently being studied in combination with IgG-based immunotherapy. In contrast, the combination with IgA-based treatment has received minimal attention. IgA antibodies have been demonstrated to more effectively attract and activate neutrophils than their IgG counterparts. Therefore, myeloid checkpoint inhibition could be an interesting addition to IgA treatment and has the potential to significantly enhance IgA therapy.

## Introduction

For many patients, cancer is a devastating disease that is caused by an unfavorable imbalance between the immune system and the tumor. Many current cancer immunotherapies attempt to restore this balance by boosting the patient’s immune system through strategies such as monoclonal antibodies (mAb), adoptive T-cell transfer, or therapeutic vaccines. mAbs are an established therapeutic tool due to their well-documented clinical activity in many different tumor types and indications. Following the approval of rituximab in 1997 for the treatment of B-cell lymphoma, many others, including trastuzumab (approved in 1998 for HER2 overexpressing breast cancer) and cetuximab (approved in 2009 for colorectal cancers) were developed (1). However, over recent years it has become apparent that clinical responses to therapeutic antibodies are often critically affected by the balance of immunostimulatory signals – typically mediated by immunoreceptor tyrosine-based activation motifs (ITAM)-containing Fc receptors (but also other molecules) – and immunoinhibitory signals mediated by a plethora of different ITIM-containing molecules (2). The term immune checkpoint blockade refers to the interference of these interactions between immunoinhibitory molecules and their ligands (3).

Identifying and blocking checkpoint molecules on T cells has evolved as a successful strategy for cancer treatment. For example, the discovery of cytotoxic T-lymphocyte-associated protein 4 (CTLA-4) and programmed cell death protein 1 (PD-1)/programmed death-ligand 1 (PD-L1) accelerated the field’s progress. Antibodies that inhibit these checkpoints, thereby preventing inhibitory signals from suppressing T cells, have been shown to improve anti-tumor responses. After the discovery of these checkpoint molecules, in 2011, the FDA approved ipilimumab, an anti-CTLA-4 blocking antibody, for patients with metastatic melanoma (4). Pembrolizumab and nivolumab, both PD-1 blocking antibodies approved for the same indication, followed in 2014 (4). Later the FDA approved atezolizumab and avelumab, both antibodies against PD-L1 (5). In the following years, the list of indications for these antibodies quickly expanded to many different tumor entities (6). However, tumor types with typically low response rates were also observed, which stimulated research into the mechanisms of antibody response and resistance (7).

Today’s inhibitory checkpoint landscape extends beyond T cells and adaptive immunity. Antibody-mediated phagocytosis of cancer cells is a primary anti-tumor response mediated by macrophages in innate immunity (8). Genome-wide CRISPR screens have identified critical regulators of macrophage-mediated antibody-dependent cellular phagocytosis (ADCP) of tumor cells. The well-known CD47 anti-phagocytic factor and genes associated with protein sialylation are among the top hits. Moreover, a novel gene, adipocyte plasma membrane-associated protein (APMAP), traditionally associated with white adipose tissue differentiation but not previously associated with phagocytosis, was discovered to strongly desensitize tumor cells to ADCP (9). Such regulators are overexpressed on cancer cells to evade immune surveillance by myeloid immune cells. For example, the binding of CD47 to signal regulatory protein α (SIRPα) on macrophages reduces their anti-tumor response, which can be restored by blocking this interaction (10). Interestingly, SIRPα and other myeloid regulators are often also expressed on neutrophils, which are the most abundant immune cell in the circulation and are also found in many tumor cell infiltrates (11).

Meanwhile, several CD47-SIRPα directed treatments are being studied in combination with tumor-directed monoclonal antibodies, which are of the human IgG1 isotype (12). While IgG1 antibodies have been found to activate neutrophils, IgA antibodies have been shown to be significantly more effective. IgA-mediated antibody-dependent cellular cytotoxicity (ADCC) by neutrophils outperforms IgG-mediated ADCC using this cell subset, and when combined with CD47 blockade, the anti-tumor capacity can be greatly increased using IgA antibodies (13, 14).

Although IgA has been proven to be particularly effective in activating neutrophils, the numerous regulators overexpressed on tumor cells indicate that antibody-driven cytotoxicity alone is insufficient to battle cancer, and that antibody therapy should be combined with checkpoint inhibition to be effective (9). This review summarizes myeloid checkpoint molecules and proposes a novel combination strategy involving therapeutic IgA antibodies.

## Myeloid Immune Checkpoint Molecules

Traditional therapeutic antibodies, such as rituximab and trastuzumab, generally rely on Fc receptor expression on effector cells to induce cytotoxic effects such as cellular activation, cytotoxicity, and phagocytosis of antibody-opsonized tumor targets (15). The primary innate myeloid population driving the anti-tumor response, likely by phagocytosis, was proposed to be IgG-complex crosslinking to FcγRIIIa on macrophages (16). Crosslinking of the IgG-complex induced phosphorylation of the ITAM tyrosine residues and led to downstream regulation of actin polymerization and activation of phagocytosis (17). Just like the inhibitory Fc gamma receptor FcγRIIb, myeloid checkpoint molecules contain immunoreceptor tyrosine-based inhibitory motifs (ITIM) with tyrosine residues that, when phosphorylated, initiate downstream signaling that suppresses phagocytosis by counteracting ITAM signaling (18).

**Figure 1:**
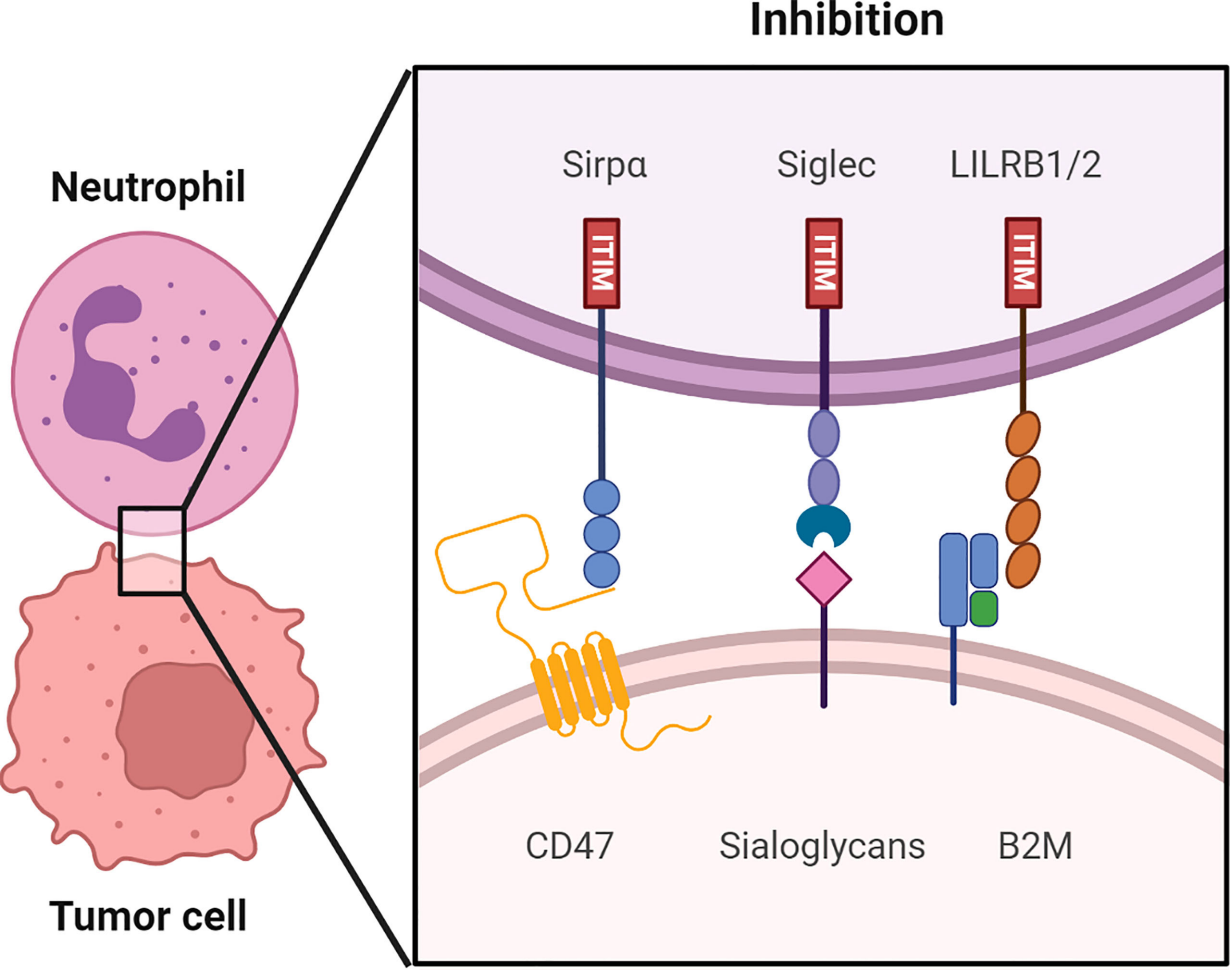
An overview of putative immune checkpoints molecules regulating myeloid cell function in human tumor microenvironments (TME). Receptor-ligand interaction including the CD47-SIRPα axis, sialoglycans-Siglec axis and HLA1 (B2M)-LILRB 1/2 axis in the immune synapse between the myeloid cells, such as neutrophils and macrophages and the tumor cells. Src family kinases phosphorylate the ITIM upon binding of checkpoint molecules to their respective receptors. The following recruitment and activation of SHP-1 and SHP-2 suppresses the anti-tumor immune responses. Consequently, tumor cells are able to evade immune surveillance.

Weissman’s lab discovered the first myeloid checkpoint molecule, the CD47-SIRPα interaction, in 2009 (19). Following that, researchers have been looking for alternative myeloid checkpoints. To date, ITIM bearing sialic acid-binding immunoglobulin-like lectins (Siglecs) expressed on myeloid cells have been discovered to bind to tumor cell ligands and inhibit immune responses in a manner similar to the CD47-SIRPα interaction. Moreover, leukocyte immunoglobulin-like receptor subfamily B (LILRB) receptors 1 and 2 on myeloid cells, were discovered to bind β2 microglobulin (B2M) exerting a similar inhibitory response in myeloid cells. The role of these myeloid checkpoint molecules (**Figure 1**) in cancer immunotherapy will be further examined.

## CD47-SIRPα Axis

One of the most studied myeloid checkpoints is the CD47-SIRPα axis. The ligand CD47, which is overexpressed on the tumor, has an Ig-like domain in its extracellular region as well as five transmembrane domains. It is ubiquitously expressed in healthy tissues, including platelets and erythrocytes, and can bind to integrins, thrombospondin-1, and signal regulatory proteins, the most relevant of which is SIRPα, since other signal regulatory proteins, such as SIRPβ and SIRPγ, bind with minimal affinity to CD47, if at all (20).

Because of its demonstrated role in immunotherapy resistance, CD47-SIRPα has received a lot of attention (21). SIRPα is a plasma membrane protein that is expressed on myeloid cells such as macrophages, granulocytes, some dendritic cell subsets, and neurons (22). SIRPα was also found to be overexpressed on natural killer (NK) cells after IL-2 activation (23). In its extracellular region, the protein has three Ig-like domains, where the NH2 terminal V-set domain is a critical CD47 interaction site (24). Furthermore, two ITIMs are present in its intracellular regions, which are phosphorylated by Src family kinases upon interaction with CD47. Subsequently, Src homology region 2 domain-containing phosphatase-1 (SHP-1) and SHP-2 are recruited and activated. These phosphatases prevent the assembly of myosin IIA at the immunological synapse, preventing phagocytosis or trogoptosis (25).

In a physiological setting, the CD47-SIRPα interaction is a critical mediator of hematopoietic cell homeostasis, most notably in erythrocytes and platelets. Healthy cells are protected from phagocytes by high CD47 expression, whereas senescent cells have low CD47 expression and are quickly cleared (20). Furthermore, recent research has discovered that functional CD47-SIRPα interaction is crucial for the survival of T-and NK cells in a steady state condition (26).

### Tumor Cells Redirect the Immune System by up Regulating CD47 Expression

**Figure 2:**
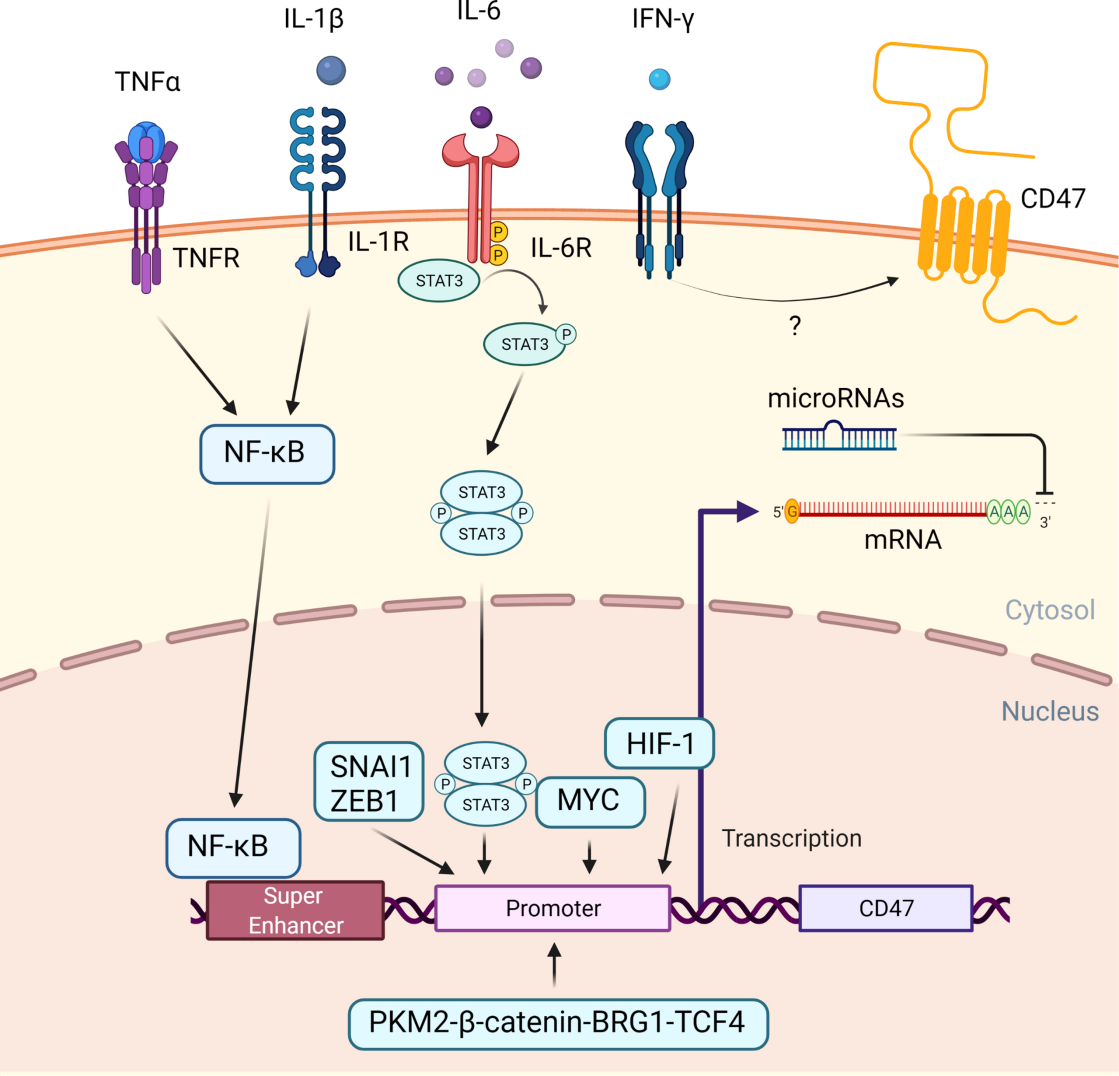
Regulation of CD47 expression in cancer cells. An overview of the mechanisms that cause CD47 overexpression in cancer. When the TNF receptor and the IL-1 receptor are activated by extracellular TNF-α and IL-1, NFκB is recruited and translocated to the nucleus, where it binds to a super-enhancer to promote CD47 expression. Extracellular IL-6 induces STAT3 signaling. Phosphorylated STAT3 complex and other transcription factors, including SNAI1, ZEB1, MYC, HIF-1, PKM2-β-catenin-BRG1-TCF4 complex enhance CD47 expression by directly binding to the CD47 promotor. Extracellular IFN-γ increased the CD47 expression, albeit the exact mechanism is unknown. Subsequently, at a post-transcription level, microRNAs could bind to the 3’ untranslated region of CD47 mRNA, causing translation to be disrupted.

In both solid tumors and hematological malignancies, such as ovarian cancer, breast cancer, multiple myeloma and Non-Hodgkin lymphoma (NHL), elevated CD47 expression is associated with a poor prognosis (19, 27–35). Several mechanisms have been proposed to explain regulation of CD47 expression in cancer cells (**Figure 2**).

Transcriptional CD47 super enhancers were found in tumor cells with a high level of CD47 expression. Moreover, additional research discovered the presence of a functional enhancer E7 upstream of the CD47 gene, with increased activity correlating with CD47 overexpression in a number of cancer cell lines. Two other enhancers, E5 at a downstream CD47 gene-associated super enhancer and E3.2 within an upstream CD47 gene-associated super enhancer, were discovered in some but not all cancer cell lines, indicating that CD47 regulation may be tumor specific (36).

While on the protein level, numerous cytokines secreted by tumor-associated macrophages (TAMs) contribute to immune evasion. Tumor necrosis factor alpha (TNF-α) promotes translocation of nuclear factor kappa-light-chain-enhancer of activated B cells (NFκB) to the nucleus in tumor cells. It binds to E5 and possibly E7 at this site, allowing bromodomain-containing protein 4 (BRD4) to be recruited to the super enhancer site and promoting CD47 gene transcription (36, 37). Co-culture of TNF-α with cancer cells at concentrations up to 100 ng/ml resulted in a maximum fourfold increase in CD47 expression due to enhanced CD47 promotor activity. This could be reversed by using an anti-TNF-α antibody-to disrupt the TNF-α – TNF receptor interaction (36, 38). Similarly, interleukin-1β (IL-1β) was shown to activate NFκB and enhanced CD47 expression in cervical cancer cells (39).

Other cytokines, such as interleukin-6 (IL-6) and interferon gamma (IFN-γ), also increased CD47 expression by activating the signal transducer and activator of transcription 3 (STAT3) pathway (32). Co-culture with these cytokines at concentrations ranging from 20 ng/ml to 100 ng/ml decreased anti-tumor ADCP of various cancer cells as a direct result of increased CD47 expression (40).

In addition, several transcription factors bind to the CD47 promotor to increase CD47 transcription. MYC, a transcription factor involved in cell proliferation, differentiation, and apoptosis, was discovered to promote tumorigenesis *via* direct binding to the promotor of the checkpoint proteins CD47 and PD-L1 (41, 42). MYC inactivation in various tumor cells down-regulated cell surface expression of CD47 (41). Similarly, recruitment of Snail Family Transcriptional Repressor 1(SNAI1), Zinc Finger E-Box Binding Homeobox 1(ZEB1), Hypoxia-inducible factor-1 (HIF-1), and the Pyruvate kinase isozymes M2 (PKM2)-β-catenin- Brahma‐Related Gene‐1 (BRG1)- Transcription Factor 4 (TCF4) complex to the CD47 promotor increased CD47 expression (43–45).

Finally, microRNAs could negatively regulate CD47 gene expression in cancer cells by degrading messenger RNA or limiting translation. Numerous microRNAs that bind to the CD47 3’ UTR and inhibit its expression have been identified. MiR-133a, for example, is downregulated in esophageal squamous cell cancer (46, 47). In T cell acute lymphoblastic leukemia, miR-708 is downregulated and sustained CD47 expression (48, 49). Other miRNAs known to influence CD47 expression include miR-155 (50), miR-200a (51), miR-192 (52), and miR-340 (53), which all act post-transcriptionally on CD47 mRNA.

### CD47 Ligation Impairs Anti-Tumor Response

The interaction between CD47 and SIRPα on macrophages inhibits phagocytosis, serving as a “don’t eat me” signal, which may counteract pro-inflammatory responses mediated by cancer therapeutic antibodies, reducing therapeutic efficacy. Targeting this axis is hypothesized to disrupt the inhibitory signal, improving the efficacy of therapeutic cancer antibody candidates targeting tumor associated antigen (TAA), such as rituximab (31). Because CD47 is expressed in both hematological and solid tumors, it is a promising and broadly applicable therapeutic target. Although CD47-specific blocking antibodies are capable of improving macrophage-mediated ADCP and neutrophil-mediated ADCC, the mechanisms underlying CD47 targeted treatment are not restricted to these functions.

Some CD47 antibodies (**Table 1**) have been reported to induce tumor cell death *via* a caspase-independent mechanism (64). Apoptosis induced by CD47 ligation appears to be epitope-dependent, as demonstrated by anti-CD47 clones MABL (60) and CC2C6 (65), but not with clone B6H12 (19, 54).

**Table 1:** The characteristics of anti-human CD47/SIRPα antibodies and proteins.

Name	Target	Isotype	Origin	Status	REF
ADU-1805	SIRPα	IgG2	Humanized	Research	(54)
ALX148	CD47	Inactive Fc domain	Fusion protein	Phase 1/2/3	(55)
AO-176	CD47	IgG2	Humanized	Phase ½	(56)
B6H12	CD47	IgG1	Mouse	Research	(57)
B6H12.2	CD47	IgG1	Humanized	Research	(19)
BI 765063	SIRPα	IgG4	Humanized	Phase 1	NCT04653142 (58),
BRIC126	CD47	IgG2b	Mouse	Research	(59)
CC2C6	CD47	IgG1	Mouse	Research	(60)
CC-95251	SIRPα	IgG1	Humanized	Phase 1	NCT05168202
Hu5F9-G4	CD47	IgG4	Humanized	Phase I/2	(61)
MABL sc(Fv)2	CD47	Sc(Fv)2	Mouse	Research	(62)
MY-1	CD47	IgG2a	Rat	Research	(63)

Moreover, treatments using CD47 antibodies can promote adaptive T-cell immune responses. Anti-CD47 therapy administered intratumorally to colon adenocarcinoma tumor (MC38)-bearing syngeneic WT C57BL/6 mice decreased tumor growth, but no effect was observed in T cell-deficient nude mice. CD8^+^ T cell depletion revealed that the therapeutic effect was mediated by cytotoxic T cells. Additionally, dendritic cells, but not macrophages isolated from the tumor microenvironment demonstrated increased cross-priming of the cytotoxic T cells (55, 56). The infiltration of CD8^+^ dendritic cells into the spleens of colon carcinoma tumor (CT-26)-bearing mice was increased following treatment with ALX148, a high affinity anti-CD47 fusion protein with blocking capacities, Moreover the dendritic cells were more activated. Increased levels of splenic effector memory and central memory CD4^+^ T cells, as well as increased numbers of central memory CD8^+^ T cells, were observed in these mice (55). In a NHL model, CD47 mAb synergized with a CD19/CD3 bispecific T cell engagers (BiTE) and activated both macrophages and T cells. It was demonstrated that the CD47 mAb required macrophages, PBMCs, and possibly others for optimal tumor clearance, as validated by macrophage depletion using liposomal clodronate or NOD/SCID mice lacking human PBMCs (57). It has been proposed that the association between CD47 on tumor cells and thrombospondin-1 on T cells acts as an immunological checkpoint, impairing T cell activation and thereby decreasing antigen-dependent killing of tumor cells by CD8^+^ T cells (58). Overall, inhibition of the CD47-SIRPα axis promotes tumor antigen cross-presentation to CD8^+^ T cells by dendritic cells, boosting the adaptive response.

### Blocking the CD47-SIRPα Axis Improves Macrophage-Mediated Phagocytosis

CD47 antibodies, such as the humanized B6H12 (often referred as B6H12.2) and BRIC126 (**Table 1**) have been shown to be effective in pre-clinical mouse models of acute myeloid leukemia (AML) (19), acute lymphoblastic leukemia (27), T-cell lymphoma (TCL) (54), and various solid tumors. After CD47 was blocked, TAMs in solid tumors could be converted to pro-phagocytic (59, 61). Interfering with the CD47-SIRPα interaction slowed tumor growth and prevented metastasis. Both B6H12.2 and BRIC126 are first generation CD47 antibodies that disrupt the CD47-SIRPα interaction while also triggering Fc-FcγR ADCC and ADCP, i.e. NK-mediated cell death, *via* an intact IgG Fc tail. Monotherapy in PDX and immunocompetent TCL mouse models using these antibodies was proven to be efficient in killing tumor cells due to their Fc effector properties, whereas antibodies with blocking properties need to be used in combination therapies (54).

Despite promising results in (pre)clinical studies, the field remains concerned about the clinical applicability of this approach. CD47 is expressed ubiquitously on somatic cells, including platelets and erythrocytes, which is of major concern for CD47 targeted therapy. Thrombocytopenia occurred spontaneously in CD47-deficient mice due to uncontrolled platelet phagocytosis. The CD47-SIRPα interaction was found to be directly involved in the regulation of macrophage uptake of CD47-negative platelets. Additionally, CD47-deficient mice develop anemia and die prematurely as a result of rapid erythrocyte clearance (62, 63, 66). As a result, CD47 must be targeted with caution. Blocking antibodies such as F(ab) fragments and Fc silent mAbs directed at CD47 were developed to overcome dose-limiting toxicity. Again, these blocking antibodies demonstrated that interrupting SIRPα signaling requires the presence a pro-phagocytic signal, as the blocking antibody alone had no effect on tumor suppression (19, 31, 67).

For example, Calreticulin and SLAMF7, are pro-phagocytic receptors that are found in a variety of malignancies but are minimally expressed on healthy cells. Calreticulin expression is increased in a range of cancers, including leukemias, bladder cancer, and ovarian cancer, whereas SLAMF7 expression is increased in predominantly hematopoietic tumor cells (68). When CD47 was blocked, Calreticulin overexpression resulted in an increase in macrophage phagocytic capability. Surprisingly, increased expression of SLAMF7 had no effect on macrophage phagocytosis (54, 68, 69). Disrupting the CD47-SIRPα downstream SHP-1 signaling in a melanoma mouse model by using mutant mice lacking the SIRPα cytoplasmic tail had no effect on tumor growth or metastasis. Tumor formation in these mice was, however, prevented when combined with an anti-gp75 antibody (28, 70). It is clear that, in addition to removing the ‘brake’, an activating cue, whether a therapeutic Fc-active antibody or another pro-phagocytic signal like calreticulin, is required for optimal immune response.

Multiple pro-phagocytic signals may be beneficial in the treatment of more advanced, resistant late-stage cancers. Blocking CD47 did, in fact, synergize with rituximab and improved macrophage-mediated phagocytosis in NHL xenotransplant mouse models (31). In a pre-clinical model of HER2^+^ breast cancer, a combination of a CD47 mAb and trastuzumab was also found to successfully augment macrophage-mediated phagocytosis (71).

### The Development of Next-Generation Antibodies

The humanized anti-CD47 antibody developed by Arch Oncology, AO-176 (**Table 1**), has the distinctive property of displaying low affinity to normal healthy cells and negligible binding to erythrocytes and platelets. The differential binding was most likely caused by the antibody’s binding epitope characteristics, which allows it to bind more potently to tumors in an acidic microenvironment (72, 73). Other novel strategies for reducing the on-target toxicity of CD47 targeting have been investigated. To reduce Fc-mediated effector functions, the humanized 5F9 (Hu5F9) antibody was placed on an IgG4 scaffold. Hu5F9-G4 inhibited the CD47-SIRPα interaction and induced phagocytosis of AML cell lines by macrophages. Furthermore, Hu5F9-G4 reduced NHL engraftment *in vivo*, but total tumor clearance was only achieved when combined with rituximab (74, 75). The on-target toxicity of these modified antibodies is significantly reduced. These agents, however, still face the issue of a large antibody sink and are restricted to combination therapies as they lack Fc-mediated effector functions.

ALX148 is a SIRPα-Fc protein engineered by fusing a modified SIRPα to an inert human IgG1 Fc portion, not capable of binding FcγR or C1q complement protein (55). Binding to the neonatal Fc receptor, on the other hand, was retained, which is beneficial for serum half-life. When compared to the WT SIRPα, it was found to have a 7000-fold higher affinity for human CD47. Because of the inert Fc portion on-target adverse reactions were reduced. *In vivo* anti-tumor responses were achieved in mantle cell lymphoma and gastric tumor models using obinutuzumab and trastuzumab, respectively. ALX148 promoted macrophage phagocytosis, induced dendritic cell activation, and promoted T cell activation in non-human primates with a favorable safety profile (55).

Another strategy to limit hematotoxicity are antibodies that target SIRPα. Several SIRPα antibodies are currently undergoing clinical trials or being researched, such as BI 765063 (NCT04653142), CC-95251 (NCT05168202), and ADU-1805 (76). These antibodies do not bind to erythrocytes and platelets and show similar efficacies *in vitro* as CD47 antibodies (76–78). However, validation in mouse models are difficult because of the human specificity. MY-1 a SIRPα antibody that binds to the NH2-terminal Ig-V–like domain of mouse SIRPα and thereby blocks the CD47/SIRPα interactions in mice. MY-1 showed no hematotoxicity in mice and improved ADCP and ADCC activity (14, 79). Moreover, the antibody also binds the extracellular region of mouse SIRPβ1 and promoted anti-tumor immunity independent of macrophage-mediated ADCP (80). SIRPβ1 lacks an ITIM motif and does not bind CD47 efficiently (81). It was found that ligation of MY-1 to SIRPβ1 promoted TNFα secretion by macrophages and suppressed tumor growth through activation of a DAP12-Syk- MAPK signaling pathway (80).

Small molecules are being investigated as a novel strategy for disrupting the CD47-SIRPα interaction. A haploid genetic screen revealed that the enzyme glutaminyl-peptide cyclotransferase-like protein (QPCTL) is required for the formation of pyroglutamate on CD47. QPCTL is an enzyme that catalyzes the conversion of N-terminal glutamine and glutamic acid residues to N-terminal pyroglutamate residues (82). The CD47 protein’s N-terminal pyroglutamate is the primary binding site for SIRPα (24). SEN177, a QPCTL inhibitor, or QPCTL knockout abolished the binding of an anti-CD47 antibody (CC2C6) that recognizes the same recognition site as SIRPα (83). Pre-treatment of MDA-MB-468 and A431 cells with SEN177 for three days, decreased SIRPα-Fc binding dose dependently and enhanced antibody-dependent macrophage-mediated ADCP and neutrophil-mediated ADCC (84). Traditional CD47 antibodies also bind to CD47 on healthy cells, leading to a decrease in the antibody’s bioavailability, also known as the antigen sink issue. Since pyroglutamate modification occurs early in the cell cycle, before reaching the cell surface, QPCTL inhibitors bypass the antigen sink problem and may not compete with physiological SIRPα (83). It has not been determined if tumor cells utilize the QPCTL enzyme to stabilize the CD47-SIRPα interaction. The QPCTL expression level did not correlate with the activation of phagocytosis in response to anti-CD47 antibodies (54).

Small molecules/peptides are an intriguing study target due to their favorable hematotoxicity profile. Both Pep-20 and RRx-001 are such small compounds that, when administered, disrupt the CD47-SIRP axis with minimal systemic toxicity (85, 86). More small molecules are likely to be discovered as a result of optimized high throughput screenings for the discovery of new CD47-SIRPα inhibitors (87, 88).

Furthermore, an anti-leishmanial drug, sodium stibogluconate, was found to improve neutrophil-mediated killing of B cell malignancies that were previously resistant to anti-CD20 IgG antibodies. The drug had no direct effect on the CD47-SIRPα axis, but was only effective when the CD47-SIRPα interaction was disrupted. Despite the fact that sodium stibogluconate inhibits the phosphatase SHP-1, the therapeutic effect was not entirely dependent on SHP-1 (89). This indicates that the CD47-SIRPα interaction is a key suppressor in the tumor microenvironment.

## Alternative Myeloid Checkpoints

### Sialoglycans and Siglecs

Many tumor cells, including colorectal, breast, ovarian, prostate, non-small cell lung cancer, and glioma, overexpress sialic acid sugar-containing glycans known as sialoglycans, which have emerged as important regulatory molecules in tumor development (90, 91).

The hypoxic tumor microenvironment, as well as the presence of oncogenic Ras, were proposed to support tumor cell hypersialylation. The 2,6-sialyltransferase enzyme (ST6Gal-I) found downstream of Ras oncogene signaling is responsible for the addition of α2,6-sialic acid to termini N-glycans (92, 93). In breast cancers, the cyclooxygenase COX-2 can increase the α2,3-sialyltransferase enzyme (ST3Gal-I) expression (94). Moreover, both ST6Gal-I and ST3Gal-(I, III and IV) overexpression was associated with poor clinical outcome in different malignancies, including myeloid leukemia, hepatocellular carcinoma and bladder cancer (95–98).

By interacting with Siglecs, sialoglycans modulates immunological responses (99). Siglecs are a family of 14-transmembrane proteins that are expressed by the majority of immune cells. They are classified into two groups: those with a conserved structural motif (Siglec-1, -2, -4, and -15) and those related to Siglec-3 (Siglec-3, -5 to -11, -14, and -16). Each Siglec has an extracellular sialic acid-binding site that recognizes sialylated proteins to which it can bind.

The majority of human Siglecs have an intracellular ITIM that is phosphorylated in response to sialoglycan binding, which results in the recruitment of SHP1/2 phosphates that inhibit downstream activation pathways (100, 101). Among the ITIM-containing Siglecs expressed on neutrophils, tumors predominantly express sialoglycan ligands for Siglec-9 and to a lesser extent for Siglec-7 (90, 91). Siglec-9 recognizes both α2,3- and α2,6-linked sialic acids at low affinity, but not α2,8-linked sialic acids. Whereas, Siglec 7 preferentially binds α2,8-linked sialic acids (102–104).

The removal of sialic acids from tumor cells using sialidase impaired their interaction with Siglecs on immune cells. The interaction of sialic acid on the tumor cell and Siglec on the immune cell results in an inhibitory immunological response mediated by activation of ITIM signaling in the tumor microenvironment (105). Although the majority of Siglec ligands remain undiscovered, certain ligands have been identified.

### HLA I-LILRB Axis

LILRB receptors, also known as ILT, LIR, or CD85, are type I transmembrane glycoproteins that have an extracellular Ig-like domain and intracellular ITIM motifs. They are expressed in human myeloid and lymphocyte cell populations. LILRB1 (ILT2, LIR-1, CD85j) and LILRB2 (ILT4, LIR-2, CD85d) are currently the best understood (106). Both receptors bind MHC class I (107), where LILRB1 only binds B2M-associated MHC class I heavy chains, while LILRB2 also binds B2M-free MHC class I heavy chains (108, 109). Both LILRB1 and LILRB2 seem to have a role in inhibiting phagocytosis by macrophages, albeit to a different extent. In one study, LILRB1, but not LILRB2, was found to inhibit macrophage-mediated phagocytosis. This could be due to the lower surface expression level of LILRB2 in macrophages (107, 110). In yet another study, LILRB2 ligation also decreased FcγR-dependent phagocytic capacity and ROS production (111).

These LILRB receptors were discovered to be active in tumor immune evasion due to their association with MHC class I. In humans the MHC Class I complex is made up of HLA α chains as well as the invariant B2M. MHC class I is ubiquitously expressed on nucleated cells and is responsible for presenting endogenous peptides to cytotoxic T cells (112). The peptide-binding cleft is formed by two membrane-distal domains (α1 and α2) that are non-covalently bound to two membrane-proximal domains (α3 and Beta 2 Microglobulin). Domains α1 and α2 interact with the T cell receptor of the cytotoxic T cell, while α3, which has a conserved Ig like domain, interacts with the CD8 co-receptor (112).

MHC Class I molecules are classified as either classical (HLA-A, B, and C) or non-classical (HLA-E,-G,-F). While LILRB1 and LILRB2 recognize the conserved α3 and B2M domains in most HLA haplotypes, HLA-G has emerged as an interesting binding partner due to its more restricted expression on tumor cells. HLA-G is mainly expressed on placental trophoblasts and thymic epithelial cells in healthy tissue and is best known for suppressing maternal immune responses (113). Tumor cells express HLA-G *de novo* to evade immune surveillance, similar to the immunosuppressive function seen in the placenta. High HLA-G expression has been found in a variety of cancers, including lung cancer (114), breast cancer (115, 116), colorectal cancer (117), gastric cancer (118), and esophageal squamous cell carcinoma (119). Furthermore, the presence of HLA-G on solid tumors has been related to poor prognosis (120). HLA-G is also expressed on hematological malignancies, although a connection between expression and tumor growth has not been established (121–124). Both LILRB1 and LILRB2 bind to HLA-G, however LILRB2 binds with a more α3 domain dominant hydrophobic interaction, confirming the B2M independency, suggesting a higher affinity association than LILRB1-HLA-G (113).

LILRB1 was discovered to interact with HLA class I and soluble HLA-G, and thereby reduce antibody-dependent NK cell induced cytotoxicity. By blocking this interaction, cetuximab-mediated ADCC was restored (120). Antibody-dependent cytotoxicity was also shown to be impaired in macrophage-mediated phagocytosis of tumor target cells (110). In this study, LILRB1 was shown to be more prevalent than LILRB2 in both healthy donor-derived macrophages and tumor-associated macrophages. These observations led to the assumption that LILRB1 is the primary mediator of MHC class I signaling in human macrophages (110).

Other LILRB members, LILRB3 (ILT5, LIR-3, CD85a), LILRB4 (ILT3, LIR-5, CD85k) and LILRB5 (LIR-8, CD85c) are orphan receptors with little information about their function (125). Since LILRB5 is not expressed on myeloid cells, it plays a minor, if any, role in myeloid immune regulation (125).

## IgA Antibodies

The majority of studies on the therapeutic relevance of myeloid checkpoint therapy have concentrated on macrophage-dependent phagocytosis. Myeloid checkpoints are typically not restricted to expression on macrophages and monocytes. Neutrophils, for example, account for the majority of white blood cells in circulation, making them an intriguing effector population. Ongoing clinical trials for CD47-targeted antibodies are focused on combining mAb IgG therapy with CD47 blocking, primarily aimed at the effect of macrophages. However, IgG antibodies can also activate neutrophils.

Neutrophils express two classical activating FcγRs, namely FcγRI (CD64) at less than 2000 molecules, and FcγRIIa (CD32a) at 30.000-60.000 molecules (126). The majority of IgG-induced neutrophil activation is mediated by FcγRIIa, the most prevalent FcγR in neutrophils (127, 128). Both IgG1 and IgG2 are able to induce neutrophil-mediated ADCC through FcγRIIA binding (129). Moreover, the FcγRIIA-H131 allotype has increased binding affinity to IgG1 and IgG2 compared to the FcγRIIA-R131 allotype, which was also reflected in the ADCC capacity (130). Engineered IgG antibody with a G236A substitution enhanced FcγRIIA binding and improved ADCC (131, 132).

**Figure 3:**
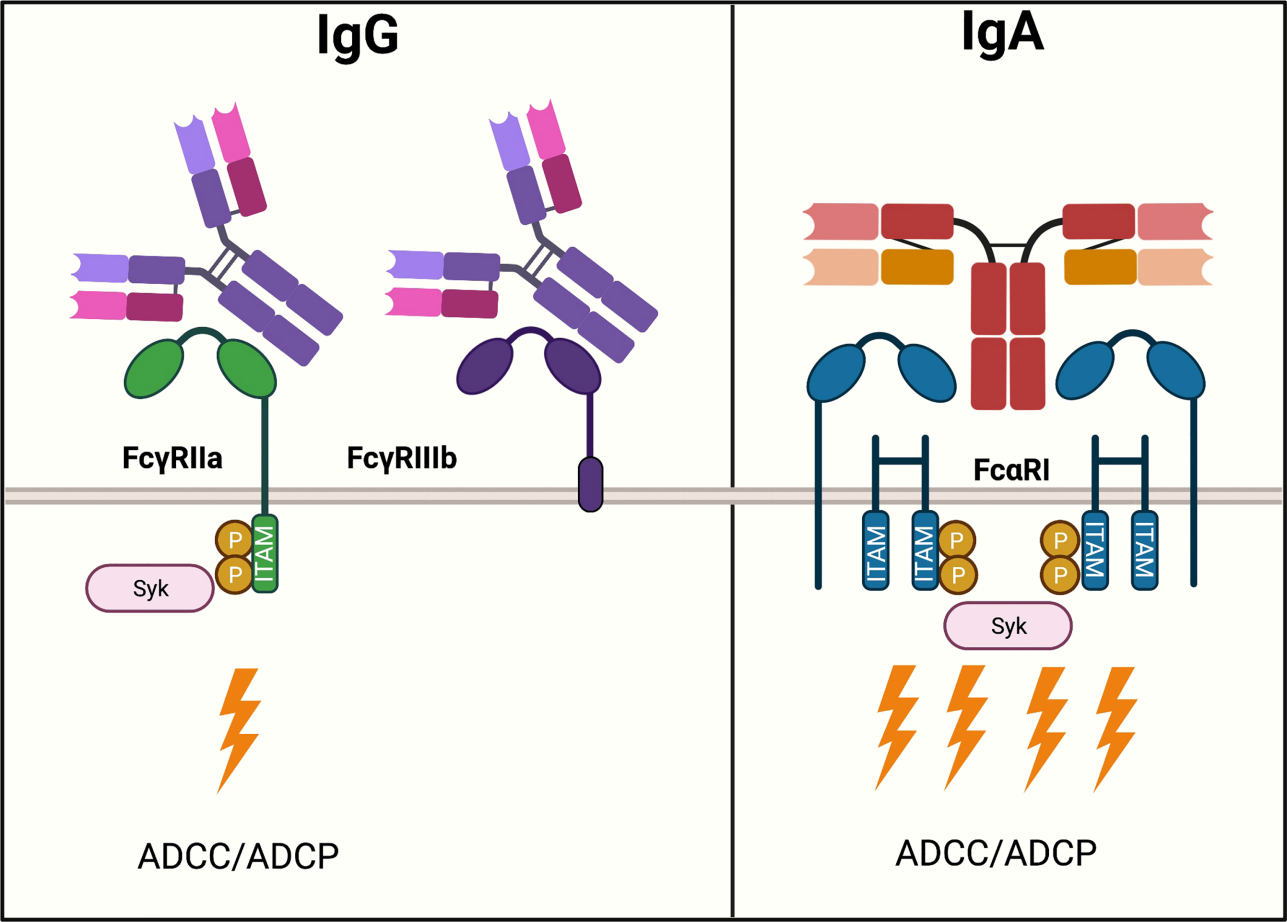
IgG- and IgA-mediated activation of neutrophils. Neutrophils express various Fc receptors, the two most abundant of which, FcγRIIa and CD16b, are important in modulating activation upon IgG ligation. FcγRIIb is expressed nearly 9-fold greater than FcγRIIa. FcγRIIa is an activating receptor that binds to IgG in a 1:1 stoichiometry and signals *via* one ITAM motif. Downstream ITAM signaling activates effector functions such as ADCC. Moreover, neutrophils express FcγRIIb, which lacks an active intracellular signaling domain and functions as a scavenger receptor for IgG. IgA binds to FcαRI expressed on neutrophils, an activating Fc receptor in a 1:2 stoichiometry. A total of four ITAMs cause a strong activation of ADCC.

Moreover, neutrophils express FcαRI (CD89) which can crosslink with IgA-complexes. Unexpectedly, IgA induced substantially more robust neutrophil activation than IgG with only about 10.000 molecules of FcαRI per neutrophil (133, 134). IgA elicited much stronger ITAM signaling compared to IgG, which was thought to be triggered due to the 1:2 stoichiometry, resulting in the activation of four ITAMs at once (**Figure 3**). (133, 135, 136) However, neutrophils also express the inhibitory receptor FcγRIIb (CD32b) and GPI-anchored FcγRIIIb (CD16b). The latter is expressed nearly 9-fold greater than FcγRIIa and lacks an active intracellular signaling domain, thereby scavenging away IgG and thus acts as a decoy receptor (137–139). FcγRIIIb does not bind IgA but competes for binding IgG with activating FcγRIIa, thereby reducing IgG Fc-FcγRIIa mediated ADCC potential (138).

IgA is the most abundant immunoglobulin produced in the human body, with a daily production rate of 66 mg/kg (140, 141).A significant amount of IgA is found on mucosal surfaces, mainly in dimeric or secretory form, where they play an important role in mucosal defense. However, IgA is also present in serum, with monomeric IgA being the second most abundant immunoglobin at 1-3 mg/ml (142). In humans two subclasses of IgA are found, IgA1 and IgA2 where the latter has two major allotypes, IgA2m(1) and IgA2m(2). Structural differences between IgA1 and IgA2 are primarily found in the elongated hinge region of IgA1, and higher number of N-glycosylation sites of IgA2. Both subclasses share a C-terminal tailpiece of 18 amino acids needed for dimerization. About 95 percent of IgAs in serum are monomeric, of which 90 percent being IgA1 and 10 percent being IgA2 (140, 143, 144).

### Signaling Mediated by IgA Fc-FcαRI

IgA antibodies bind to FcαRI, a type 1 transmembrane receptor expressed on neutrophils, eosinophils, monocytes, macrophages, and some dendritic cell subsets (140, 145). IgA-complexes associate with a dimer FcR gamma chain containing two ITAMs, followed by tyrosine phosphorylation of the ITAMs. This then acts as a docking site for the tyrosine kinase Syk, which, when activated through calcium release, activates downstream targets such as PI3K, phospholipase C-γ and NADPH oxidase (146–148). Furthermore, ITAM activation enhances phagocytosis, oxidative burst, ADCC, and cytokine release (134, 144, 149). In soluble form, both IgA isotypes have low binding affinity to the FcαRI with a K_a_ of 10 (6) M^-1^, but this is increased by 30-fold when IgA is immobilized (136, 140). Two domains on IgA, the CH2 and CH3 domains, have been identified as critical FcαRI binding sites (136).

Whereas ADCC by NK cells mediated by IgG induces apoptosis of target cells *via* perforin and granzymes, neutrophilic cytotoxicity is mediated by different mechanisms. Neutrophil mediated cytotoxicity is distinguished by an irregular neutrophil shape and a swarming effect towards the tumor cell (150). In attempting to phagocytose larger tumor cells, neutrophils adhere and spread around their target, a process previously known as frustrated phagocytosis (151). Recently, this mechanism of neutrophil-mediated effector function responsible for superior killing of cancer cells was identified as trogoptosis. It is a mechanism that involves active disruption of the cell plasma membrane which results in lytic cell death (152). The first lytic events were discovered to occur within 20 minutes, indicating that neutrophils are very efficient at IgA-mediated cell death (133).

IgA antibodies have been recombinantly engineered against a variety of targets, including CD20, EGFR, GD2 and HER2 (153–156). cetuximab IgA variants were developed and shown to block the EGFR ligand-binding domain, inhibit EGFR phosphorylation, and inhibit EGF-induced cell growth in the same way as IgG1 cetuximab (157, 158). When these IgG antibodies were converted to IgA antibodies, their Fab-mediated activities were unaffected (159, 160). Upon analyzing antibody-mediated cytotoxic functions, it was demonstrated that IgG1 antibodies primarily induced ADCC by peripheral blood mononuclear cells (PBMCs) and to a much lesser extent by polymorphonuclear leukocytes (PMNs). IgA variants, on the other hand, induced ADCC mainly by PMNs but not by PBMCs in an FcαRI-dependent manner (154). Furthermore, IgA appears to play a role in macrophage-mediated phagocytosis (161–164). However, when monocytes/macrophages were used as effector cells, the difference was less pronounced, indicating that PMNs are the most important effector cell population for IgA mediated killing. Indeed, at an E:T ratio as low as 5:1, IgA was able to induce PMN-mediated ADCC. In experiments with IgA2-EGFR complexed with A431 cells using whole leukocytes, mimicking physiological conditions, IgA2 outperformed IgG1 in ADCC (157–159). Myeloid cell numbers can be easily increased by GM-CSF and G-CSF and have been shown to improve IgA anti-tumor effects. However, it was not required for effective IgA mediated kill (153, 162, 165).

Although promising, preclinical studies with IgA therapeutic antibodies have been challenging for decades. One of the limitations include the short half-life of IgA compared to IgG. IgA lacks the binding site for the FcRn, resulting in a significantly shorter serum half-life. Additionally, hepatic clearance of exposed terminal galactose by the asialoglycoprotein receptor (ASGPR) reduces the half-life (166, 167). However, through extensive antibody engineering, the IgA antibody has been improved in producibility and stability (153, 154, 157–160). Moreover*, In vivo* studies have been limited by the absence of FcαRI in mice and the rapid clearance of IgA. Pre-clinical models to study IgA were developed by the generation of a functional hFcαRI transgenic mouse, described here (168).

The first demonstration of the anti-tumor response of IgA *in vivo* was shown in an EGFR model using FcαRI transgenic mice, using both IgA1 and IgA2 EGFR antibodies (162). The typical fast clearance of the IgA antibody was resolved by repetitive dosing to match the serum levels of IgG. Multiple doses of 50 μg IgA2 EGFR antibody restricted tumor growth in an A431 lung carcinoma SCID model in a FcαRI dependent manner (162). Furthermore, in A431 cell-based short-term intraperitoneal (i.p.) models, i.p. treatment with multiple doses of 50 μg IgA suppressed outgrowth while increasing macrophage and neutrophil influx. However, the anti-tumor response appears to be primarily Fab-dependent rather than ADCC-dependent. In a short term i.p. syngeneic C57BL/6 model with Ba/F3-EGFR cells IgA2 EGFR induced cytotoxicity, but it was primarily mediated by macrophages/monocytes (162). IgA2 induced a cytotoxic response that lasted longer than cetuximab in a long-term immunocompetent C57BL/6 model (162). An engineered variant of IgA2, termed IgA2.0, against EGFR inhibited outgrowth as well. IgA2.0 has improved pharmacokinetics and has been found to be beneficial in long-term *in vivo* models (153). IgA2.0, was more effective than IgA2 in the i.p. model, most likely due to its improved serum stability (153). Following that, other models produced demonstrated comparable results for other targets, including CD20 (13, 154), HER2 (155, 159) and GD2 (169). To summarize, IgA antibodies have demonstrated efficient anti-tumor responses in various tumor models expressing various tumor target antigens.

## Combining Myeloid Checkpoint Inhibition With IgA Antibodies

### The Role of Neutrophils in Myeloid Checkpoint Inhibition

**Figure 4:**
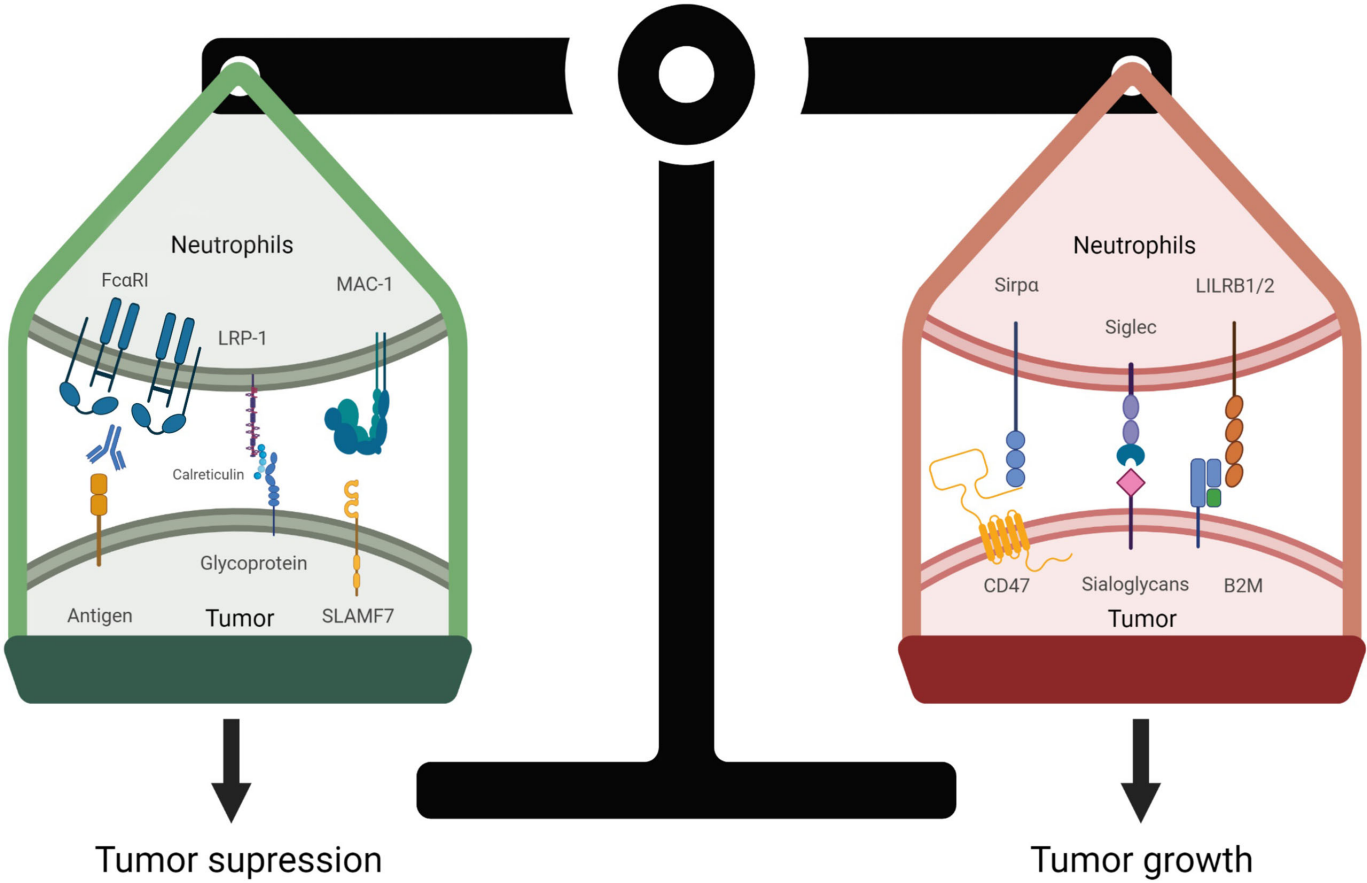
Regulation of neutrophil-mediated tumor cell death. The balance of pro-phagocytic and anti-phagocytic signals determines the fate of the tumor cell. Pro-phagocytic signals are elicited by Fc receptor engagement to IgA-opsonized tumor cells, resulting in Fc activation and phosphorylation of downstream ITAM tyrosines. Calreticulin is tethered to the cell surface by membrane glycans and interacts with lipoprotein receptor-related protein 1 (LRP1) receptor expressed on neutrophils. Likewise, SLAMF7 (CD319) binds to macrophage-1 antigen (MAC-1, α_M_β_2_), these interactions promote tumor cell killing by neutrophils. In contrast, overexpression of CD47 on tumor cells interact with SIRPα to inhibit neutrophil activation. Similarly, if expressed, the sialoglycans-Siglec axis, and HLA1 (B2M)-LILRB axis if expressed decrease immune responses, allowing tumor cells to evade immune surveillance.

Killing of target cells by an anti-CD47 mAb was reduced in the absence of macrophages, showing that macrophages mediate the majority of therapeutic effectiveness (170). However, because neutrophils play such a minor role in IgG immunotherapy, this effector population is often overlooked. Nevertheless, recent research has highlighted the importance of neutrophils in myeloid checkpoint inhibition. In a variety of neuroblastoma cell lines, knocking down CD47 or blocking SIRPα on neutrophils increased the neutrophil-mediated ADCC induced by dinutuximab (anti-GD2 antibody). Blocking the CD47-SIRPα interaction also increased ADCC capacity in primary patient-derived neuroblastoma spheroid cells as long as GD2 expression was sufficient, once again highlighting the relevance of combination therapy (**Figure 4**) (171, 172). Furthermore, anti-CD47 monoclonal antibodies were shown to work synergistically with trastuzumab to improve neutrophil ADCC against breast cancer cell lines (28, 152). Cetuximab-opsonized A431 cells, as well as trastuzumab-opsonized A431 cells, demonstrated enhanced neutrophil-dependent tumor killing in response to CD47-SIRPα inhibition (152, 173). The role of neutrophil killing in CD47 checkpoint inhibition was subsequently examined *in vivo*, where disruption of the CD47-SIRPα axis significantly reduced the metastatic load in the liver and was revealed to be dependent on neutrophil killing (152). When the CD47-SIRPα axis was blocked by an anti-human SIRPα antibody, KWAR23, not only macrophages but also neutrophils infiltrated the tumor in a human Burkitt’s lymphoma xenograft. Neutrophil depletion resulted in tumor growth, suggesting that neutrophils, like macrophages, also play an anti-tumor role (174).

To support the role of granulocytes, in NHL models, the synergy between rituximab and anti-CD47 occurred independently of NK cells or complement (31). Furthermore, in melanoma, CD47 may protect NK cells from chronic inflammation, and CD47 deficiency resulted in splenic NK cell exhaustion (175). Since clinical IgG antibodies primarily act through NK cell-mediated ADCC of tumor cells and, to a lesser extent, interact with macrophages and neutrophils, it is debatable whether IgG-mediated tumor killing is the best strategy (176).

### IgA Synergizes With CD47 Targeted Therapy

We hypothesize that the observed effects of myeloid cells upon CD47 blockade could be enhanced even further by the use of IgA antibodies, which primarily recruit neutrophils and macrophages thereby potentially benefiting the most from CD47-SIRPα targeted therapy.

Previous research has shown that targeting CD47-SIRPα in combination with IgA therapeutic antibodies is beneficial. *In vitro* studies showed improved IgA-mediated ADCC by PMNs upon disruption of the CD47-SIRPα interaction in SKBR3 and A431 cells, either by blocking SIRPα or knocking out CD47 in the tumor cell line. PMN-ADCC mediated by IgA2 trastuzumab and IgA2 cetuximab in CD47KO SKBR3 and CD47KO A431 cells, respectively, improved ADCC more than 10-fold when compared to IgG equivalents (14). The beneficial role of IgA in CD47 blocking therapy was translated into *in vivo* findings as well. In an A431 hFcαRI Tg xenograft mouse model, CD47 KO A431 cells were subcutaneously injected in one side of the flank and WT A431 cells in the other flank, and mice were treated with either IgA anti-EGFR, cetuximab or control. Only the IgA treated mice showed reduced tumor size for the CD47 KO tumor. Improved anti-tumor response was also observed in a shorter syngeneic i.p. Ba/F3-HER2 model, where consistent with previous data, IgA outperformed trastuzumab in the absence of CD47-SIRPα signaling. The CD47-SIRPα axis was disrupted at the neutrophil site using an anti-SIRPα blocking antibody (clone MY-1), demonstrating various strategies for disrupting the inhibitory interaction. In this model, IgA-HER2 was responsible for an enhanced influx of granulocytes to the tumor, whereas trastuzumab did not improve the influx of granulocytes (14). In a similar mouse model, but a different approach to interrupting the CD47-SIRPα signaling, QPCTL deficient Ba/F3-HER2 cells that lack pyroglutamate on CD47, were effectively killed by neutrophils directed to the tumor by IgA-HER2, in a similar fashion to CD47KO Ba/F3-HER2 cells (83). These studies consistently show that IgA synergizes with CD47 targeted therapy and outperforms IgG in a variety of tumor cell lines and targets in both short and long term mouse models.

### The Immunosuppressive Function of Siglecs in Myeloid Cells

While the sialoglycan-siglec and HLA1 (B2M)-LILRB1/2 axis have rarely been explored in combination with IgA antibodies, they have been found to be ITIM-mediated, comparable to the CD47-SIRPα interaction. These alternative checkpoint receptors have been shown to inhibit FcγR-mediated IgG responses in monocytes and macrophages. Because of their expression on neutrophils and the inhibition mediated by ITIM signaling, these inhibitory receptors are likely to modulate FcαRI signaling in a similar fashion.

The immunosuppressive function of Siglecs in neutrophils has been well described for Siglec-9 in both bacterial infections and tumor immunology (177, 178). Accordingly, Siglec-9 agonists were reported to prevent neutrophil activation and NETosis in SARS-CoV-2, where NETosis is undesirable due to respiratory damage (179). In a tumor co-culture *in vitro* experiment, Siglec-9 ligation on neutrophils resulted in decreased activation as measured by ROS generation (90). Similarly, siglec-9 binding to glycophorin A on erythrocytes inhibited NET formation and ROS production (180). The activation of Siglec-9 suppresses both caspase-dependent and caspase-independent neutrophil-induced apoptosis of tumor cells (181).

Siglec-E (the mouse homolog of Siglec-9) expression allows for preclinical mouse studies. Siglec-E was reported to have an immunosuppressive effect on neutrophils in an acute inflammatory model. Ligation of Siglec-E reduced the influx of neutrophils, by inhibiting CD11b signaling (182). This inhibitory response was controlled by activation of NADPH oxidase and the production of ROS by Siglec-E (183). The inhibitory role of Siglec-9/Siglec-E was also verified in an *in vivo* MC38 tumor intravenous (i.v.) model, where Siglec-E knock out mice had decreased lung metastasis (90). Siglec-E is found on infiltrating neutrophils, macrophages and DCs in B16 and MC38 tumormodels (184). Furthermore, Siglec-9 is also expressed on macrophages and has been demonstrated to reduce TNFα secretion while increasing IL-10 production (185). Surprisingly, contrary to the immunosuppressive findings, Siglec-E knock out mice demonstrated increased M2 macrophage polarization in later stages of tumor growth, indicating that the stage of cancer should be considered when targeting Siglec-E/9 (90).

To generate a humanized immunocompetent mouse model, Siglec-E was knocked out and human Siglec-7 and -9 were introduced (184). B16 tumors were inoculated s.c. in this model to assess the antibody tumor response. When treated with the gp76 antibody, Siglec-E KO mice had less tumor outgrowth than Siglec-7^+^/Siglec-9^+^ Siglec-E KO mice, indicating that Siglec-7 and Siglec-9 decrease the antibody response (184). Although it is evident that tumor cell hypersialylation has an immunosuppressive function, little is known about the Siglec-ligands.

These ligands have been investigated as potential novel immunotherapy targets. Previously, it was discovered that Mucin 1 (MUC1) and MUC16, which are frequently overexpressed in adenocarcinomas, bind Siglec-9 (186, 187). MUC1 binding to Siglec-9 on macrophages induced calcium flux, which activated the MEK-ERK pathway. This resulted in the macrophages adopting an more immunosuppressive TAM phenotype (188). Similarly, MUC 16 is highly expressed in ovarian carcinoma where siglec-9 binding mediates inhibition of anti-tumor immune responses (187, 189).

Moreover, lectin galactoside-binding soluble 3 binding protein (LGALS3BP) or Mac-2 binding protein was recently found as a Siglec-9, -5 and -10 ligand. However, Siglec binding is likely not restricted to LGALS3BP and MUCs, and there may be additional unidentified ligands (186). Recombinant LGALS3BP inhibited both spontaneous and Lipopolysaccharides (LPS)-induced neutrophil ROS generation, indicating decreased neutrophil activation. Furthermore, when neutrophils were co-cultured with LGALS3BP knock out HT-29 tumor cells, neutrophil mediated apoptosis was higher than in WT HT-29 cells (190).

Siglec-7 is also expressed at low levels on neutrophils, but its immunosuppressive function is less well characterized. One of the Siglec-7 ligands was recently identified as GD2, which is overexpressed on neuroblastoma cells. By activating neutrophil-mediated ADCC and disrupting the Siglec-7-GD2 axis, GD2-targeted mAbs effectively induced killing of neuroblastoma cells (172).

### The Immunosuppressive Function of LILRB in Neutrophils

LILRB expression on neutrophils seems to be phase dependent. Mainly LILRB2 is expressed on the cell surface of healthy peripheral neutrophils in a steady state condition. While there have been contradictory findings on the expression of LILRB1, proteomic analysis has verified LILRB1 expression on neutrophils (191). LILRB1 expression was increased in primed neutrophils, indicating that LILRB2 controls immune responses throughout the middle and late activation phases of the neutrophil lifecycle, avoiding overactivation (111).

In neutrophils, LILRB2 has shown to reduce neutrophil cytotoxicity. Both antibody-dependent phagocytic functions and neutrophil ROS production were inhibited by HLA-G interaction (111).

Despite the fact that LILRB3 and LILRB4 are orphan receptors, they remain interesting receptors to investigate in the context of myeloid cells. LILRB3 is expressed on neutrophils and suppresses the formation of ROS *via* FcαRI in infectious diseases. After one hour of incubation on an anti-LILRB3 or isotype control coated plate, neutrophils were activated *via* FcαRI crosslinking. In this study, ROS production was decreased when neutrophils were incubated in wells coated with anti-LILRB3. Following that, phagocytosis mediated by FcαRI was evaluated using IgA1-opsonized microparticles. The phagocytic uptake of microparticles was decreased in LILRB3-blocked neutrophils. Similarly, LILRB3 impaired IgA-mediated phagocytosis of bacteria (*S. capitis*) (192). Therefore, we should not limit our attention to LILRB1 and 2.

Moreover, crosslinking of a different family member LILRB4 using immobilized anti-LILRB4 monoclonal antibody, inhibited FcγRI-dependent phagocytosis and TNFα production *via* monocytes through phosphatase recruitment (193, 194). Interestingly, LILRB4 expression was found on PMN- Myeloid derived suppressor cells (MDSCs) but not on PMNs obtained from healthy control donors in one study of 105 non-small cell lung cancer patients, and expression was found to be inversely related to patient survival (195). These findings suggest that LILRB4 may play a role in neutrophil suppressive progression.

## Strategies to Combine IgA Antibody Treatment With Myeloid Checkpoint Inhibition

**Figure 5:**
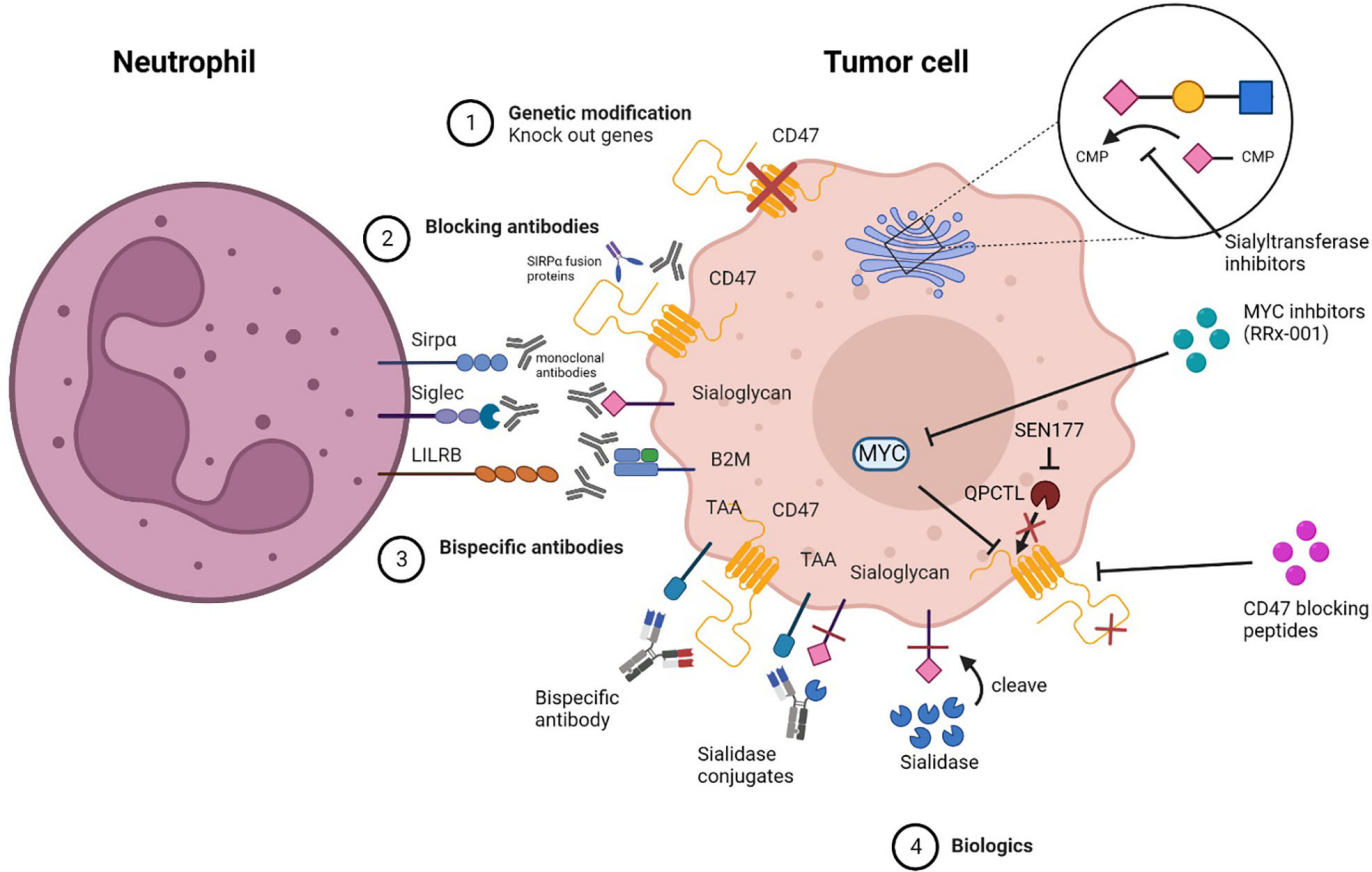
Strategies for inhibiting myeloid checkpoints. 1) Genetic knock out of target genes involved in the inhibitory pathway. 2) Specific blocking of target checkpoint molecules with mAbs or soluble ligand-Fc fusion proteins to inhibit receptor binding and checkpoint axis activation. 3) Bispecific antibodies that target both TAA and checkpoint molecules simultaneously to avoid off-target side effects.4) Biologics that alter the structure of the target protein, preventing it from binding to the receptor, or that inhibit expression or block the target protein.

As a result of checkpoint-related advancements in the IgG field, we’ve acquired a number of ways for checkpoint inhibition in IgA treatment. Since comparable approaches may be used, the switch to IgA therapy is straightforward (**Figure 5**).

A straightforward strategy for proof-of-concept experiments is to knock out target genes of interest. In CD47 studies, tumor cells can be effectively knocked out, resulting in improved anti-tumor response (173). Similarly, essential enzymes in the sialic acid metabolism pathway can be knocked out to reduce sialylation of surface glycans on tumor cells. One of these enzymes is UDP-N-acetylglucosamine 2-epimerase/N-acetylmannosamine kinase (GNE) (196).

Antibodies that bind to checkpoint molecules and block their activity can also be considered to prevent the suppressive interaction. Several antibodies have been developed to interrupt the CD47-SIRPα axis, including anti-CD47 mAbs, anti-SIRPα mAbs, and SIRPα-Fc fusion proteins (**Table 1**). Moreover, anti-Siglec-7 antibody clone Z176, S7.7 and 1E8 have been successfully shown to bind Siglec-7 (90, 184). To target Siglec-9, antibody clone 191240, E10-286 and mAbA has been tested in several studies (177, 178, 184). Furthermore, antibodies against HLA Class I [clone W6/32 (110)], LILRB1 [clone GHI/75 (110) and BND-22 (197)] LILRB2 (clone 27D2) (197) have been shown to inhibit the immunosuppressive activities of the HLA/LILBR axis and enhanced anti-tumor activity.

Small molecules and enzymes provide a novel approach to checkpoint modulation. SEN177, a small molecule glutaminyl cyclase inhibitor, for example, has been found to interfere with QPCTL function and prevent pyroglutamate synthesis on CD47, interrupting binding of SIRPα to CD47 (83). RRx-001, an inhibitor of MYC downregulates CD47, while another small molecule Pep-20 targets CD47 to disrupt the interaction with SIRPα (85, 86). Sodium stibogluconate, albeit not directly targeting the CD47-SIRPα axis, synergized with CD47 blockade and improved anti-tumor response (89).

Furthermore, cleaving surface sialic acid residues with sialidase is a frequent method for modulating the sialoglycan/Siglec interaction (198). A different approach is to target sialyltransferases, which catalyze the conversion of sialic acid residues to oligosaccharide chains by using cytidine monophosphate N-acetylneuraminic acid (CMP-Neu5Ac) as the donor. Hypersialylation can be prevented by inhibiting the sialyltransferases (199).

Finally, several strategies are presented dependent on the intended target. Small molecules and antibodies with clinical promise have received increased attention throughout the years. The majority of treatments, however, have not been clinically studied and are still in the early stages of research.

### Bispecific Approaches

Combination therapy with monovalent IgA antibodies and CD47 targeted antibodies has limitations, such as the antigen sink problem that traditional anti-CD47 antibodies suffer from (200). Similarly, combination therapy with novel checkpoint molecules will likely face the same challenge. Bispecific antibodies (bsAbs) have gained recognition as a novel format of antibody in recent years, and we believe they can potentially fill the gap left by anti-checkpoint and TAA-antibody combination therapies. Bispecific antibodies have a greater affinity for dual antigen-expressing cells than for single antigen-expressing cells, resulting in a more avid interaction.

The potential of bispecific TAA/CD47 targeting antibodies, have been demonstrated by several studies. The novel HuNb1-Ig4 antibody demonstrated a lower affinity for RBCs than the anti-CD47 antibody Hu5F9-G4. The affinity for RBC was reduced much further when developed into a bispecific antibody and linked to the C-terminus CH3 domain of rituximab. When compared to the combination treatment of rituximab and HuNb1-IgG4, the bispecific CD20/CD47 HuNb1-IgG4 significantly lowered tumor volume *in vivo* (201). However, it is unclear whether this rituximab modification will affect the ADCC capacity.

Another approach is the dual variable domain immunoglobulin (DVD-Ig)format to generate a CD20/CD47 bispecific antibody (202). Using an amino acid linker, the variable domain of one antibody was engineered on the N-terminal variable domain of the other antibody. The CD47 variable domain was placed in the inner position because steric hindrance was expected to reduce affinity. Indeed, the affinity of CD47 in the bispecific antibody format was reduced by 20-fold when compared to the affinity of CD47 mAb, whereas the affinity of CD20 appeared to be unaffected. When incubated with RBCs, the binding assay confirmed simultaneous binding to CD47 and CD20, as well as preferential binding to dual antigen-expressing cells. Furthermore, the bsAb was shown to increase phagocytosis in the same way that anti-CD47 mAb and rituximab combination therapy did. Additionally, in a subcutaneous Raji NSG mouse model, the CD20/CD47 DVD-Ig reduced tumor burden in a manner comparable to combination therapy (202).

Moreover, anti-CD47/CD19 (71) or anti-CD47/MSLN (72) antibodies were generated in the κλ body format, which has the human IgG1 isotype (203). They differ from conventional IgG1 in that they have two different light chains, one kappa and one lambda paired to the IgG1 heavy chain. The kappa light chain in the κλ body format targets CD47, while the lambda light chain targets either CD19 or MSLN. To prevent undesired binding from TAA-negative cells, the CD47 arm has a lower affinity. When tested, the κλ bsAbs demonstrated selective binding for dual antigen-expressing cells, tumor killing *in vitro*, and were superior to combination therapy with monovalent antibodies. *In vivo*, bsAbs inhibited tumor growth in the same way that the combination treatment did. These bsAbs were found to be at least as effective in mediating phagocytosis as the monovalent antibodies combined, with no undesired binding to erythrocytes or platelets, making them potential clinical candidates (204, 205).

Sialylation does not occur exclusively on the tumor’s surface. To avoid unfavorable side effects, it is critical to selectively remove sialoglycans from tumor cells while leaving healthy cells undisturbed. However, targeting sialoglycans may involve a slightly different approach. Antibody-enzyme conjugates could provide a method for removing sialoglycans from tumor cells specifically. In one study, a trastuzumab-sialidase conjugate demonstrated specific sialic acid cleavage on HER2 positive SKBR3 cells and had no effect on the HER2 negative MDA-MB-468 cells. Furthermore, when compared to trastuzumab monotherapy, the trastuzumab-sialidase conjugate demonstrated increased NK-cell mediated ADCC against a variety of HER2 expressing cell lines, making this a promising tool for avoiding on-target side effects while maintaining improved anti-tumor capacities (105).

Glycoproteins and glycosaminoglycans are large proteins found in the glycocalyx of both tumor and immunological cells. Not surprisingly, the glycocalyx has been shown to inhibit phagocytosis through steric and electrostatic hindrance. As a result, removing this barrier and revealing tumor targets may improve tumor killing. The removal of mucins from tumor cells improved phagocytosis (206). Combination strategies, in which the glycocalyx of the tumor cell is first stripped, followed by targeting other checkpoint molecules in combination with IgA treatment, could be an effective and novel strategy.

## Concluding remarks

IgA antibodies are an emerging novel strategy for antibody therapeutics that, due to their unique mode of action, hold promise for use in tumors infiltrated by neutrophils. These tumors often have a poor prognosis with currently available therapies (207) – supporting the need for new approaches. Because myeloid cells are recruited, combining CD47-SIRPα checkpoint inhibition with IgA antibodies might be a good method for targeting resistant tumor cells. Similarly, we believe that blocking other myeloid checkpoint molecules addressed in this review could improve IgA-mediated tumor responses. Combining IgA treatment with checkpoint inhibitors such as those described in **Figure 5** is proposed.

Alternatively, a bsAb IgA antibody directed against a TAA and a checkpoint molecule could be used to circumvent some of the limitations of monovalent antibody-based combination therapy. We are confident that techniques comparable to those used to generate IgG-based bsAbs can be utilized to create novel IgA-based bsAbs as long as the modifications do not interfere with FcαRI signaling. However, the efficacy of such bsAbs is unclear and needs to be further investigated. These developments could improve current therapies that use identified myeloid checkpoint molecules, while also highlighting the potential of myeloid checkpoints to accelerate the discovery of more inhibitory molecules.

## Author Contributions

CC, GT, and JL contributed to the concept of the review. CC visualized and wrote the first draft of the review. All authors contributed to manuscript revision, read, and approved the submitted version.

## Funding

This work was supported by a grant of The Dutch Cancer Society (KWF Kankerbestrijding) - Project: 11944. ML and TV are supported by the Clinical Research Unit CATCH-ALL funded by the Deutsche Forschungsgemeinschaft - Project: 444949889.

## Conflict of Interest

The authors declare that the research was conducted in the absence of any commercial or financial relationships that could be construed as a potential conflict of interest.

## Publisher’s Note

All claims expressed in this article are solely those of the authors and do not necessarily represent those of their affiliated organizations, or those of the publisher, the editors and the reviewers. Any product that may be evaluated in this article, or claim that may be made by its manufacturer, is not guaranteed or endorsed by the publisher.

## Glossary

**Table:**

ADCC	Antibody-dependent cellular cytotoxicity
ADCP	Antibody-dependent cellular phagocytosis
AML	Acute myeloid leukemia
APMAP	Adipocyte plasma membrane-associated protein
B2M	β2 microglobulin
BiTE	Bispecific T cell engager
BRD4	Bromodomain-containing protein 4
BRG1	Brahma‐Related Gene‐1
BsAb	Bispecific antibody
CD47	Cluster of differentiation 47
CTLA-4	Cytotoxic T-lymphocyte-associated protein 4
DVD-Ig	Dual variable domain immunoglobulin
GNE	UDP-N-acetylglucosamine 2-epimerase/N-acetylmannosamine kinase
GPI	Glycosylphosphatidylinositol
HCC	Hepatocellular carcinoma
HIF-1	Hypoxia-inducible factor-1
IFN- γ	Interferon gamma
IL-1β	Interleukin-1β
IL-6	Interleukin-6
ITAM	Immunoreceptor tyrosine-based activation motif
ITIM	Immunoreceptor tyrosine-based inhibitory motif
LGALS3BP	Lectin galactoside-binding soluble 3 binding protein
LILRB	Leukocyte immunoglobulin-like receptor subfamily B
LPS	Lipopolysaccharides
mAb	Monoclonal antibody
MDSC	Myeloid derived suppressor cell
MUC1	Mucin 1
NFκB	Nuclear factor kappa-light-chain-enhancer of activated B cells
NHL	Non-Hodgkin lymphoma
NK cell	Natural killer cell
PBMC	Peripheral blood mononuclear cell
PD-1	Programmed cell death protein 1
PD-L1	Programmed death-ligand 1
PKM2	Pyruvate kinase isozymes M2
PMN	Polymorphonuclear leukocytes
QPCTL	Glutaminyl-peptide cyclotransferase-like protein
ROS	Reactive oxygen species
SHP1/2	Src homology region 2 domain-containing phosphatase-1/2
Siglec	Sialic acid-binding immunoglobulin-like lectin
SIRPα	Signal regulatory protein α
SNAI1	Snail Family Transcriptional Repressor 1
SOCS3	Suppressor of cytokine signaling 3
ST6Gal-1	2,6-sialyltransferase 1
STAT3	Signal transducer and activator of transcription 3
TAA	Tumor associated antigen
TAM	Tumor-associated macrophages
TCF4	Transcription Factor 4TCL T-cell lymphoma
TME	Tumor microenvironment
TNF-α	Tumor necrosis factor α
ZEB1	Zinc Finger E-Box Binding Homeobox 1
