# Corrigendum: Targeting myeloid checkpoint molecules in combination with antibody therapy: A novel anti-cancer strategy with IgA antibodies?

**DOI:** 10.3389/fimmu.2022.1017924

**Published:** 2022-09-13

**Authors:** Chilam Chan, Marta Lustig, Niklas Baumann, Thomas Valerius, Geert van Tetering, Jeanette H. W. Leusen

**Affiliations:** ^1^Center for Translational Immunology, University Medical Center Utrecht, Utrecht, Netherlands; ^2^Division of Stem Cell Transplantation and Immunotherapy, Department of Medicine II, Christian Albrechts University Kiel and University Medical Center Schleswig-Holstein, Kiel, Germany

**Keywords:** IgA, myeloid checkpoints, neutrophils (PMNs), cancer immonotherapy, immune checkpoint, antibodies, CD47-SIRPalpha axis, macrophages

In the published article, there was an error in **Figure 5** as published. The mitochondrion was misplaced in this figure. The corrected **Figure 5** and its caption appear below

**Figure 5:**
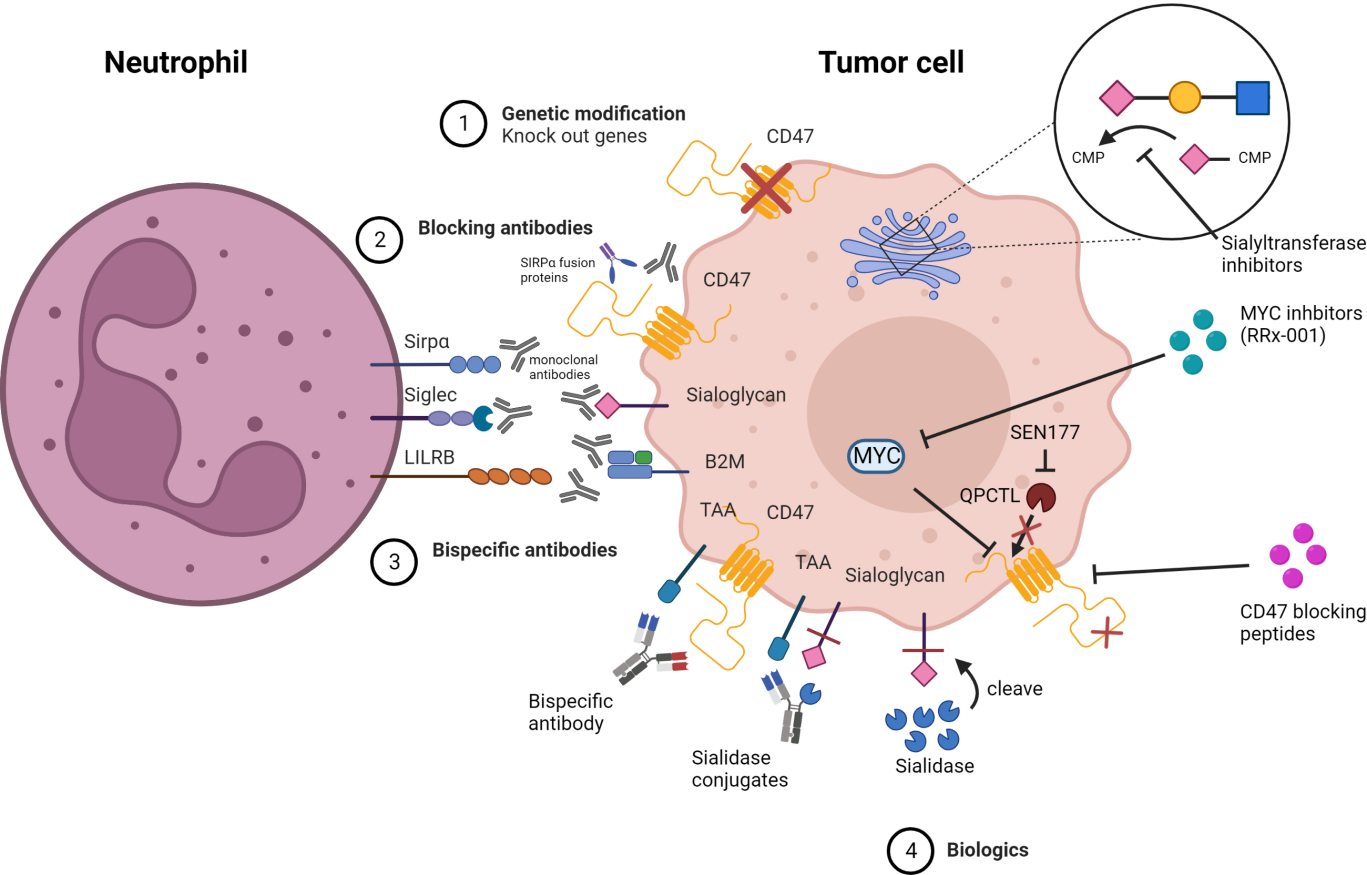
Strategies for inhibiting myeloid checkpoints. 1) Genetic knock out of target genes involved in the inhibitory pathway. 2) Specific blocking of target checkpoint molecules with mAbs or soluble ligand-Fc fusion proteins to inhibit receptor binding and checkpoint axis activation. 3) Bispecific antibodies that target both TAA and checkpoint molecules simultaneously to avoid off-target side effects.4) Biologics that alter the structure of the target protein, preventing it from binding to the receptor, or that inhibit expression or block the target protein.

The authors apologize for this error and state that this does not change the scientific conclusions of the article in any way. The original article has been updated.

## Publisher’s note

All claims expressed in this article are solely those of the authors and do not necessarily represent those of their affiliated organizations, or those of the publisher, the editors and the reviewers. Any product that may be evaluated in this article, or claim that may be made by its manufacturer, is not guaranteed or endorsed by the publisher.

